# Socioeconomic determinants of rural women's desired fertility: A survey in rural Shaanxi, China

**DOI:** 10.1371/journal.pone.0202968

**Published:** 2018-09-13

**Authors:** Jieqiong Wei, Jianhong Xue, Duolao Wang

**Affiliations:** 1 College of Economics and Management, Northwest A&F University, Shaanxi, China; 2 Liverpool School of Tropical Medicine, Liverpool, United Kingdom; Austrian Academy of Sciences, AUSTRIA

## Abstract

There has been evidence demonstrating that China has had a persistently low and below-replacement level fertility since early 1990s, causing concerns of a rapidly aging population and sustainability of the Chinese economy. To avoid adverse effects of excessively low fertility, the Chinese government has recently changed its family planning policy from "one-child policy" to "two-child policy." Nonetheless, the effectiveness of the newly initiated two-child policy is questionable if women's average desired number of children or desired fertility for their lifetime is below the threshold fertility allowed by the two-child policy. Therefore, this study argues that it would be interesting and pertinent to know women's fertility desires under the circumstances of no policy restrictions and understand major factors that may affect their desired fertility. Based on a multi-stage stratified cluster sampling survey with 2,516 women respondents in rural Shaanxi, this study tries to estimate desired fertility of rural women and evaluate the impact of important socioeconomic factors on their desired fertility. The results of this study reveal that the average lifetime desired fertility for rural women of childbearing age in Shaanxi is about 1.71, below the total fertility rate at the replacement level. The findings of this study suggest that women's marriage age, the pecuniary costs of having children, women's income forgone for having children, and social security benefits available for rural residents at retirement age, are significantly and negatively related to desired fertility. However, rural women's cultural views towards fertility are significantly but positively related to their desired fertility. This study further confirms that China has entered an era of low fertility, and thus, any policy restrictions on fertility may no longer be necessary. Instead, government programs which support childbearing and childrearing are needed to prevent excessive low fertility and rapid aging of the population.

## Introduction

Historically, China had a very high fertility rate due to its cultural tradition of family's obsession with male heirs to extend family lineage and endorsement of early marriage, resulting in a huge and increasing population size [1]. Shortly after the establishment of the People’s Republic of China in 1949, Chinese women's fertility rates remained high for the period of 1950–1975. The average total fertility rate (TFR) was about 6 for the period of 1950–1970, and around 5 for the period of 1970–1975 [2, 3].

It was a general belief that a huge and increasing population in China had led to a high man-to-land ratio, resulting in the so-called "high level-equilibrium trap," causing historical stagnation of the Chinese economy and the withering of the Chinese Civilization [4–6]. For the sake of economic development, the Chinese government first initiated a family planning program in early 1970s, called "later, longer, and fewer" (*wan-xi-shao*) campaign, advocating later marriage, longer spacing, and fewer children [7, 8].

In 1979, the government took this a step further and introduced the one-child policy, allowing only one child per couple, to deliberately control high fertility and slow down the rapid increase of the Chinese population [9, 10]. The policy was equipped with economic and administrative incentives for one-child families but penalties for couples with out-of-plan births [8, 11]. While the policy was strictly enforced in urban areas, it was to a certain extent relaxed or up to local discretion in rural China [9]. As a result, the TFR has kept slightly over 1.0 since 1979 for urban women excluding floating immigrants [12], whereas the figure was about 2.7 for rural women for 1980s and 1990s [10].

On average, the TFR for the country as a whole remained between 2.2 and 2.3 for the period of 1980–1990, just above the replacement level 2.1 per woman [3]. However, studies have revealed that in 1992, TFR for Chinese women had dropped to 2.0, which was below the replacement level for the first time [3, 13]. Subsequent studies have shown a decreasing and persistent below-replacement level TFR for Chinese women since then, with the lowest number of 1.05 recorded in 2015 [14]. Therefore, there are growing concerns that a persistently low fertility rate will lead to rapid population aging and a shortage of labor in the workforce in the near future, adding great pressure on China's social security, healthcare, and economic sustainability [15, 16].

To avoid the adverse effects of excessively low fertility, the Chinese government has taken action by modifying the one-child policy. In the Third Plenary Session of the 18th Central Committee of the Chinese Communist Party in November 2013, a new family planning program, namely the "selective two-child policy" (*dan-du-er-hai-zheng-ce*) was introduced [17]. Under this program, couples in which either the wife or the husband, or both, came from a single child family are allowed to have two children. Because the follow-up evaluation of the 2013 selective two-child policy has revealed low second child registration for couples that meet the criteria for two children [18], the government further relaxed its restrictive policy measures, and a newly revised two-child policy was formally initiated and became effective since January 1^st^, 2016, known as "universal two-child policy" (*quan-mian-er-hai-zheng-ce*) [19], allowing all Chinese couples to have two children.

The one-child policy was undoubtedly largely responsible for the decrease in fertility rate, especially in 1980s and 1990s. However, the experience of fertility decline in the developed world indicates that the economic transition from pre-industrial stagnation to modern economic growth was generally accompanied by a demographic transition from high to low fertility [20]. It is noteworthy that China's economic reform and modernization began in 1978, about the same time as the initiation of the one-child policy.

Following nearly four decades of rapid economic development and industrialization, China has had unprecedented economic growth and social changes. Consequentially, the impact of these socioeconomic changes on fertility in the country has been very significant, and the contribution of socioeconomic factors to the decline of fertility has far exceeded that of the one-child policy. As some recent surveys suggest, the introduction of the two-child policy could cause only a marginal increase in fertility for women of childbearing age. For instance, a large survey by Ma & Gu has demonstrated that by the end 2014, about 11million couples met the criteria for the 2013 selective two-child policy, but only about 9% of them (around 1 million couples) registered for the second child [18]. Gu's further study has suggested even lower second child registration rate for couples who meet the 2013 selective two-child policy criteria: only around 5% registration rate for those couples in most of the regions in the country [21]. The marginal increase in fertility is mainly based on urban women's response to the change of the one-child policy. However, the enforcement of the newly-initiated universal two-child policy could have no effect on rural women's fertility decision because the "one-child" policy had already been relaxed for rural residents since the 1980s [22].

Given the fact that rural women had traditionally been the major source of high fertility, it would be meaningful to know whether, in the absence of any policy restrictions, rural women would like to have more than two children. Therefore, this study focuses on the following two questions: (i) What is the desired fertility (desired number of children) of rural women under the circumstance of no policy restrictions? (ii) What are the major factors that may affect rural women's desired fertility in the context of socioeconomic changes? Answers to these questions could have potential implications to devise appropriate policies in preventing China from being a rapid aging population.

To address these two questions, a multi-stage stratified cluster sampling survey was conducted in rural Shaanxi province from 1^st^ of May to 31^st^ of October in 2015. Based on the survey of 2,516 women respondents of childbearing age, this study collected data on women's desired fertility and its socioeconomic determinants. The result of this study and its implication in devising more effective population policy are discussed. Section 2 of this paper includes a review on economic theory of fertility, important socioeconomic factors that may affect individuals' fertility decision, as well as the changes of socioeconomic contexts in rural China. Section 3 explains the data and methods used for analysis, and the results of this study are presented in section 4. In section 5, concluding remarks and recommendations to China's population policy are made.

## Economic theory of fertility and the changing context of fertility in rural China

In the current literature, several theories have been developed to explain the underlying forces that affect society's fertility transition and individual's fertility intention [23, 24]. Although these theories are diverse, they can be used to elucidate society's fertility changes or individual's fertility decision in given socioeconomic conditions.

In the context of "natural fertility," married couples do not consciously limit the number of their children [25]. Describing "fertility control" as parity-specific behavior to restrict fertility, Henry believed that the evolution from natural fertility to controlled marital fertility is critical to the transitions from high levels of fertility to low levels of fertility in societies. Similarly, Walle argued that in pre-transition societies, people do not have clear concept of desired family size or consciously think about family size [26]. Therefore, sustained declines in marital fertility in societies have been considered the results of conscious planning by couples to limit their fertility after reaching their desired family size [27].

These arguments suggest that conscious planning is essential to regulate fertility. Such a proposition may be strengthened by the explanation of the European fertility decline from 1870 to 1930 caused by the dissemination of ideas and knowledge of the means of fertility control [23]. The availability of the knowledge and effective means of fertility control can be seen as an important precondition for making conscious planning practical. However, Notestein argued that contraceptive methods have been widely used in societies for centuries but was not widely used until the incentive for birth control becomes strong in the age of industrialization, suggesting that economic consideration becomes increasingly important to fertility decision as economic development reaches certain stage [28].

Coale summarized three necessary conditions for the decline of marital fertility: (i) social context that allowed for fertility planning to be based on conscious choice; (ii) the availability of information about effective means of fertility control; and (iii) clear economic benefits of fertility control [23, 29]. Coale's outline of these three conditions provides us a basis for analyzing fertility regulation for given historical and contemporary socioeconomic contexts.

In primitive societies, with no conscious planning about family size and no effective means of fertility control, fertility was mainly regulated by biological factors. Based on the fact that the fertility for pre-transition or high-fertility societies is well below the biological maximum, some scholars have argued that sociobiological factors were the major force to regulate fertility without effective means of fertility control (e.g., [30–32]).

Other studies have contended that social norms or cultural values are influential to regulate fertility, and they are thus used to explain the difference of fertility behavior among societies (e.g., [33–35]). Nonetheless, the origins of the social and cultural influences on fertility were not further explained, and empirical testing of these propositions remains a challenge [23]. In modern societies, especially industrialized ones, economic considerations become increasingly important for family decisions with more liberated conscious planning and broad availability of modern means of fertility control. All of this suggests that as societies become progressively more developed, conscious planning on desired fertility becomes much more prevalent for family decision making, with decisions being based increasingly on economic incentives and constraints.

In order to understand individuals' incentives and constraints in fertility decisions, Becker's seminal contribution to economic analysis of fertility [36] and subsequent works by economists (e.g.,[37, 38]) have provided theoretical foundations to analyzing fertility from the perspective of cost-benefit analysis. As Werding noted, economic theory of fertility offers a unique approach to analyzing relevant motives and outcomes of fertility behavior [39]. Based on a rational-choice paradigm, children are considered durable consumption goods, production goods or long-term investment goods, and thus, raising children could have significant impacts on the well-being or "utility" of parents in terms of costs and benefits, or costs and returns [23, 36]. To maximize their wellbeing ("utility") of having children, parents may have their ideal or desired number and preferred sex-combination of children in mind.

On the cost side of having children, there are direct and indirect costs of childbearing and childrearing. The direct costs include monetary expenditure on both healthcare of the mother and the child during childbearing and children’s cost of living (e.g., food, clothing, healthcare, education and other extra household items) before a certain age. The indirect cost of children generally refers to the "opportunity costs" of childbearing and childrearing, mainly including women's education and income forgone through time spent on both childbearing and childrearing [39].

On the benefit or return side, there are also direct and indirect utilities of having children. Viewing children as consumption goods, having children, like the consumption of many other goods, directly increases the utility of parents [36]. Children, as investment goods, may make future contributions to the lifetime income of their parents [40]; and returns to the investment in children accumulating at old age takes not just the form of monetary transfers, but also the form of personal care and attention to the parents [39, 41].

However, it has been argued that the direct economic utility of having children is often low and the indirect utility is usually embedded in social beliefs, for example, "marriage is for procreation" or that "children make a marriage" [42, 43]. Given that actual costs and returns to having children are fully revealed only once the child has been born, and that the utility of having them can be in the form of social beliefs or norms, reliable indicators of perceived costs and benefits of having children can be developed to understanding desired fertility or actual fertility decisions (e.g., [39, 43]).

With such an approach, various socioeconomic forces that may influence individual's desired fertility or actual choice of fertility can be classified into two categories: factors that affect the costs of having children and factors that represent the benefits of having children. Of course, the applicability of economic theory and approaches, as Werding has pointed out, is based on if choice is technically possible and if required freedom of choice is permitted [39]. Therefore, socioeconomic factors that affect desired fertility or actual fertility choice should be comprehended in given contexts.

For thousands of years, China was largely an agricultural society. Its large scale industrialization and urbanization is only a recent phenomenon. As China’s industrialization process started to accelerate over the last couple of decades, the weight of economic consideration on an individual's fertility decision has significantly increased due to tremendous changes in socioeconomic contexts.

Before the economic reform in 1978, China was notably less developed. Although there was no clear policy restriction on individuals’ fertility decisions before early 1970s, the knowledge of modern means of fertility control were limited and they were not widely available to individuals in a context of nationwide poverty and economic crises. Even if couples exercised conscious planning on their family size, their desired fertility may not be realized due to lack of effective contraceptive methods.

Significant changes in the context of fertility occurred in 1979 when the one-child policy was introduced. Under the policy, couples in China did not have the freedom of choice in terms of fertility decision. However, various modern means of contraception, including intrauterine device (IUD) insertions for women, sterilization, and abortion, have become widely available since the introduction of the one-child policy [44]. Now, with the relaxation of the one-child policy, individuals have more freedom to make their own fertility decisions. Because that both income and living expenses have had large increases due to rapid and large scale economic development and growth, the effects of socioeconomic factors are gaining great importance on individual's fertility decision. Therefore, the changes in fertility context for the last four decades in China had a huge impact on both the costs and benefits of having children, and consequently, an individual's desired and actual fertility.

Firstly, policy restriction on fertility has driven up both the direct cost and indirect cost of children. Since the enforcement of the one-child policy in China, most urban couples only have one child and rural couples, on average, have about two children. Families with fewer children pay closer attention to not only the quality of children but also the personal safety of them [45, 46]. Consequently, Chinese households spend much more on childcare, foods, healthcare, education and many other household items related to childbearing and childrearing. Parents and grandparents have paid much more attention and time to their child or children at the expense of their own education or other opportunities due to concerns about personal safety and the quality of their children. The increase in both direct and indirect costs of children may affect women's desired and actual fertility.

Secondly, the rapid industrialization and economic development provides women with much more opportunities for employment and education, thus increasing the opportunity costs of having children. In late 1970s, at the beginning of the economic reform, rural residents mainly worked in small-scale farming. As industrialization and urbanization accelerated, many rural residents started to work in other industries and earn a much higher wage than in the agriculture sector [47], with increasing numbers of rural women participating in the workforce [48]. Meanwhile, rural women may demand for education due to higher returns to human capital in the age of industrialization. Hence, income and education forgone by a woman could also be important parts of their calculations in having children.

Thirdly, the wide availability of employment and other income opportunities as well as improvements made in social security may change an individual's perceived benefits of having children. Historically, there was a tradition in which the elderly, especially those in rural China, expected to receive financial support and personal care from their children at advanced age, since social security was largely unavailable to rural residents. Such returns from having children can be perceivably seen from the social norms in China, "bring up sons for one's old age" (*yang-er-fang-lao*). Such a belief has changed in urban China because urban residents have been receiving pension from either the state-run enterprises or collective welfare programs for a few decades. As Cheng & Maxim pointed out, urban pension programs have been one of the most important factors causing the decline of China's urban fertility [49]. However, to rural residents, similar social security programs have become available only very recently, with much lower levels of coverage. Although there are no uniform social security programs in rural China, the recent availability of social security programs to rural residents may also have an important impact on their desired fertility.

To a certain extent, economic variables can be calculated or assessed. However, parents are able to determine the cost of their children by spending more or less on each child [36, 39]. Also, because of many risks and uncertainties that exist in the process of childbearing and childrearing, both the costs and benefits of having children can be assessed in perceived values [43]. In addition, from the perspective of new institutional economics, social customs and norms as informal institutions may affect fertility choices much like other constraints do [39]. In fact, social norms in a traditional Chinese context mainly highlight the benefits of having more children. For instance, "more sons, more happiness" (*duo-zi-duo-fu*), "life is more complete with both a son and a daughter" (*er-nü-shuang-quan*), "bring up sons for one's old age" (*yang-er-fang-lao*), and "having children is for extending family lineage" (*chuan-zong-jie-dai*). These social norms may represent traditional beliefs on either noneconomic "utility" or perceived benefits of having children, and thus, have a considerable influence on desired fertility. Therefore, understanding how individuals hold these traditional values towards fertility can also be helpful in comprehending the variations of desired fertility among individuals.

## Materials and methods

### Variables and hypotheses

The primary objectives of this study were to evaluate rural women's desired fertility level, and to identify its influencing factors. Therefore, the main outcome variable is "desired fertility". Based on the discussion of economic theories of fertility and the current context of China’s rural fertility, we argue that the following socioeconomic factors may have important impacts on rural women’s desired fertility, including women’s age at the time of survey, age at first marriage, education, women's income, number of siblings, direct cost of child, women's view on traditional culture towards fertility, and social security coverage available to the elderly after retirement age. The definitions of the variables and argument for the hypotheses corresponding to the independent variables are provided below.

#### Desired fertility

It refers to the desired or ideal number of children that a woman likes to have in her lifetime. In this study, desired fertility is the dependent or outcome variable.

#### Age

It is women’s actual age at the time of the survey. There was a general trend that the younger generation of women would rather have fewer children than the older generation of women. Also, we expect that the older the age the lower the desired fertility. Therefore, age reflects both the cohort and the age effects.

***Hypothesis 1*: *women’s age is positively related to their desired fertility***.

#### Age at first marriage

It means women’s actual age at the time of first marriage. Since women’s age at first marriage determines the time span for marital fertility, the earlier the first marriage, the more children women may desire, but the later the first marriage, the fewer children women may desire.

***Hypothesis 2*: *women' age at first marriage is negatively related to their desired fertility***.

#### Education

It refers to women’s level of education. In existing literature, education has been widely considered one of the major socioeconomic determinants of fertility (e.g., [52–54]). Two major arguments have been presented in various studies regarding the effects of education on fertility. One is that education may increase the opportunity cost of having children. As Miller indicated, the motivation of having children is negatively related to the level of education because the completion of education itself and opportunities associated with it promote activities competitive with childbearing and childrearing [24]. Another argument is that highly educated women are more likely to substitute child number with child quality [55, 56]. While these two arguments suggest a negative relationship between the motivation of having children and women's level of education, some empirical evidence has indicated positive associations between women's level of education and their fertility intentions [57]. Considering fertility intention an important channel through which education affects fertility, Testa argued that the association between fertility intentions and education is not necessarily the same as the association between actual fertility and education [57]. Although highly educated women intend to have more children than less educated women, studies have found that highly educated women ultimately have fewer children than intended [58]. In fact, some other studies have revealed that more highly educated women would like to revise their fertility intention downwards than less educated women [59, 60]. These all imply that the effects of women's education on desired fertility are complex, especially for given socioeconomic contexts.

***Hypothesis 3*: *women's level of education is either positively or negatively related to their desired fertility***.

#### Number of siblings

It refers to the total number of siblings of a woman respondent and her husband. Studies have found that parental fertility positively affects children’s fertility (e.g. [62–64]) and that women with more siblings are likely to have more children (e.g., [65]). Scholars argued that the positive relationship between the family sizes of successive generations could be produced by four mechanisms (e.g., [63]). One is that parental orientation, desires and norms toward family size could be learned by their children during socialization. Second, parents are a prime source of knowledge of birth control. Third, role relationships that young adults try to recreate in the family to some extent depend upon the number of children in that family. Finally, natural fertility or fecundity can be transmitted through genetic mechanisms. Hence, one would expect that women with more siblings are likely to have higher desired number of children [63].

***Hypothesis 4*: *number of siblings is positively related to women's desired fertility***.

#### Women's income

In existing literature, scholars have argued that the relationship between income and number of children could be either positive or negative. From the point of view that individuals could obtain direct pleasure from having and raising children [36], the positive relationship between income and number of children may be implied. However, a large body of literature provides two different views for the negative relationship between income and number of children. One view focuses on the tradeoff between the quality and quantity of children, suggesting parents with higher income value children’s quality and thus reduce the number of children for their given income constraint [56]. The other view acknowledges that an important part of the opportunity cost of having children is women's income forgone during childbearing and childrearing, indicating that higher income mothers are expected to have fewer children because fertility is more costly to them (e.g., [61]). Although there could be a positive relationship between income and fertility among men or in the case of incentive payment to increase fertility, with unprecedented economic and social development in China for nearly four decades, many rural women have not only high expectations for the quality of their children but also more job opportunities to earn a high wage outside of agriculture [45, 47]. Therefore, the substitution effect between child quality and quantity of children and income forgone for having children could reduce rural women’s desired fertility.

***Hypothesis 5: women's income is negatively related to their desired fertility***.

#### Direct costs of child

In the economic theory of fertility, the costs of children are argued to be one of the major factors that affect the demand for children [39]. Empirical evidence has suggested that national differences in fertility are being driven by national differences in child costs, and that women do take into account the perceived costs of bearing and raising children while making a decision about their fertility [66]. The costs of children include the costs of childbearing and childrearing with both direct and indirect costs. Since risks and uncertainty are involved in the long process of childrearing, this study only considers the major direct costs of a child as a proxy for the overall direct costs.

***Hypothesis 6: direct costs of child are negatively related to women's desired fertility***.

#### Traditional culture

It refers to women's views on traditional culture towards fertility. In traditional culture, social beliefs or norms towards fertility are generally considered an important factor on peoples’ fertility decision [39]. As mentioned in the previous section, social norms towards fertility in the Chinese cultural setting all emphasize the benefits of having more children. Therefore, women with more influence of traditional culture towards fertility would like to have more children.

***Hypothesis 7: women who are more influenced by traditional cultural beliefs or social norms towards fertility have a higher desired fertility***.

#### Social security

It means the social security coverage available to the elderly in survey regions. In societies in which there is no social security program to cover the elderly at advanced age, individuals may depend mainly on their grown-up children to provide them with financial support and personal care [68]. Nonetheless, the availability of social security to the elderly may reduce the perceived benefit or return from having more children. Some empirical studies have demonstrated that increases in social security coverage have a negative effect on fertility [49, 69]. In rural China, there has been no social security program for the elderly until recently. Since there are variations of the monthly payment to the elderly in different villages, one would expect that the higher the amount of money women expect to receive from the social security program after retirement age, the lower the desired number of children.

***Hypothesis 8*: *social security coverage for the elderly is negatively related to women’s desired fertility***.

### Survey sampling and measurements

To assess the desired fertility and factors that may affect an individual's desired fertility choice in rural China, a multi-stage stratified cluster sampling survey in rural Shaanxi province was conducted from 1^st^ of May to 31^st^ of October in 2015. The targeted population was married women of childbearing age (20–49) with a rural household identification (*nong-cun-hu-kou*) in rural Shaanxi during the survey. Shaanxi is geographically and culturally divided into 3 areas, North Shaanxi (shan-bei), Guan-zhong Plain (guan-zhong), and South Shaanxi (shan-nan) [50, 51]. These 3 areas have a total of 11 regions (all city-administered). From the 11 regions, we selected Baoji, Xi’an, and Weinan to represent Guan-zhong Plain, Yan’an to represent North Shaanxi, and Hanzhong to represent South Shaanxi. All 60 counties or equivalent districts under these selected 5 regions constitute the first level of sample frame. These 60 counties were then grouped into 3 types (I, II, and III) according to GDP per capita, disposable income per capita, and average rural household income. Subsequently, for each of the 5 regions selected, one of each type of county (or county-equivalent) was randomly selected. As a result, a total of 15 counties (or equivalents) constitute the second level of sample frame. From the sample frame, 15 vernacular villages (one from each of these 15 counties) were randomly selected. All women, age from 20 to 49, were selected by cluster sampling.

Questionnaires of this study were distributed to all women of childbearing age in each of these 15 villages. Of a total of 3,041 copies of the questionnaire issued, 2,516 were completed (with a response rate of 82.74%) and used as the sample for analysis. Before the survey was conducted, the purpose of the study was clearly explained to each respondent, and oral consents were obtained from all participants. Trained personnel carried out the survey with face-to-face interviews based on the structured questionnaire. Ethics review approval was obtained from Ethics Review Committee at Northwest A&F University in Yangling, Shaanxi, China.

Data for measurements of the variables are all from the survey. In order to know “desired fertility” or the ideal number of children, we simply asked our women respondents the question, "how many children would you like to have in your lifetime if there were no policy restrictions?" For the variable “age,” women respondents’ actual age at the time of our survey was recorded. Also, for “age at first marriage,” women respondents’ actual age at the time of first marriage was asked and recorded. For the variable “education,” we classified women’s education into three levels: "elementary or below," "middle-school," "high-school or above". The amount of money that women respondents earned in the previous year was used as the measure of “women's income”. In our survey, the total number of siblings that a women and her husband have were simply recorded to measure the variable, “number of siblings”.

The measurement of “direct costs of child” is more complex. We used the costs of healthcare and nutrition enhancements for the mother, as well as the costs of food, healthcare, childcare, education and additional household items for the child from age 0 to 6 years old to measure “direct costs of child.” Women respondents were asked to provide an assessment on the amount they have spent (experienced mothers) or expect to spend (expected mothers) on their child. Although the ways of assessment are not the same for experienced and expectant mothers, the indicators (or items) listed in the survey to assess the costs are exactly the same for both. Because the actual costs of children change overtime, variation in the assessment of costs between experienced and expectant mothers can be found in addition to some unavoidable measurement errors due to different ways of assessment. Also, some variation may arise because parents can adjust some child-related costs to their income constraints and preferences on child quality accordingly.

To evaluate how traditional culture affects women's desired fertility, women respondents were asked to provide their own judgment on the extent that they believe in the following four widely disseminated social norms in Chinese society: (i) "more sons, more happiness" (*duo-zi-duo-fu*); (ii) "life is more complete with both a son and a daughter" (*er-nü-shuang-quan*); (iii) "bring up children for one's old age" (*yang-er-fang-lao*); and (iv) "having children is to extend family lineage" (*chuan-zong-jie-dai*). The Likert 5-point scale was used for the question "How strongly do you believe in each of the following old sayings on fertility?" An index score was then calculated based on the answers to each of them with a range from 4 to 20. Then, using the method of fuzzy pattern recognition [67], the variable, traditional culture, is grouped into 3 categories: weakly influenced, moderately influenced, and deeply influenced.

To assess whether the availability of a rural social security program has had an effect on desired fertility or not, this study uses expected monthly financial support (in Chinese Yuan) for the elderly as a proxy for social security.

### Statistical methods

It is believed that desired fertility (desired number of children) follows a Poisson distribution. So Poisson regression model was used to identify the determinants of desired fertility. Given the dispersed nature of the data distribution, this study used the generalized Poisson model that can accommodate both over- and under- dispersion [70–74]. The distribution of the generalized Poisson model has a probability density function given by

$$f\left(y_{i},\lambda_{i},\alpha \right)={\left(\frac{\lambda_{i}}{1+\alpha \lambda_{i}}\right)}^{y_{i}}\frac{{\left(1+\alpha y_{i}\right)}^{y_{i}-1}}{y_{i}!}\exp\left(-\frac{\lambda_{i}\left(1+\alpha y_{i}\right)}{1+\alpha \lambda_{i}}\right)$$ (1)

*y*_*i*_ = 0,1,⋯ and *λ*_*i*_ = exp(*x*_*i*_*β*), where *x*_*i*_ is a (*k*−1) dimensional vector of variables including age, age when first married, women's education level, women's income, number of siblings, direct costs of children, traditional culture, social security, and *β* is a *k* dimensional vector of regression parameters. For more details on the generalized Poisson regression model, the reader can refer to Famoye [70].

The mean value and variance of the generalized Poisson model is

$$E\left(y_{i}|x_{i}\right)=\lambda_{i}=\exp\left(x_{i}^{T}\beta \right)$$ (2)

$$Var\left(y_{i}|x_{i}\right)=\lambda_{i}{\left(1+\alpha \lambda_{i}\right)}^{2}$$ (3)

If *α* = 0 then the generalized Poisson model reduces to the Poisson model, satisfying the mean value equal to variance; If *α* > 0 then variance is greater than mean value, over-dispersion problem can be resolved; If *α* < 0 then mean value is greater than variance, under-dispersion problem can be fixed.

We used the generalized Poisson model for two main reasons. Firstly, the desired fertility is the number of children that women desire to have in their lifetime, its range of value is very small, and the value is nonnegative integer. In addition, its distribution is neither normal nor continuous, but is dispersed and skewed. Secondly, the variance of data that we collected is far less than its expected value. In such circumstances, generalized Poisson model is a better fit to accommodate both over- and under- dispersion than normal Poisson model [70].

Analysis was done in two steps: univariate analysis was performed in the first step to assess the individual effect whereas multivariate analysis was performed in the second step to assess simultaneously the effects of various predictors on desired fertility. Incidence rate and its 95% confidence interval together with p-value were derived from the Poisson model.

Descriptive statistics were calculated to summarize outcome variable and independent variables: mean, standard deviation, minimum, and maximum were used to describe a continuous variable and percentage values were used to describe a categorical variable. All statistical analyses were performed using SAS 9.4.

## Results

### Distribution of women's desired fertility and descriptive statistics

Table 1 shows the distribution of desired fertility among 2,516 women respondents. The mean desired fertility for all those 2,516 women in rural Shaanxi is about 1.71, far below the replacement level fertility of 2.1. 30.64% of the women revealed that they would like to have only one child in their lifetime under the circumstance of no any policy restrictions, 67.93% of them indicated that two is their desired number of children, only 1.03% of them specified three as their desired number of children, 0.32% of them picked four as their desired lifetime fertility, and 0.08% of them (only two individuals in the sample) indicated that they would like to have no children at all.

**Table 1:** Distribution of desired fertility among women of childbearing age in rural Shaanxi (N = 2,516).

Desire Fertility	Frequency	Percentage
0	2	0.08%
1	771	30.64%
2	1,709	67.93%
3	26	1.03%
4	8	0.32%
Total	2,516	100.00%
Mean	1.71	
Standard Deviation	0.5	
Minimum	0	
Maximum	4	

Variables included in the regression are described in Table 2. The average age of women of childbearing age is 36.46 years. The mean of rural women’s first marriage age is 22.93 years. The data shows that 30.45% of the women had received a "high school or above" level of education, 38.99% of them had a "middle school" education, and 30.56% of them had an "elementary or below" education. The average number of siblings that women and their spouses had is around 4; the mean yearly income of women was around 17,100 Chinese Yuan; the average direct costs of a child before age 6 in rural Shaanxi is around 18,400 Chinese Yuan; the average monthly social security payment that an elderly could get after age 60 was about 122.92 Chinese Yuan. The data also indicate that 14.03% of the women were weakly influenced by traditional culture towards fertility, 65.70% of them were moderately influenced, and 20.27% of them were deeply influenced.

**Table 2:** Description of independent variables in the regression models (N = 2,516).

Variable	Mean or No. (%)	Standard Deviation	Minimum	Maximum
Age (Year)	36.46	7.43	20	49
Age at First marriage (Year)	22.93	2.17	17	35
Education (Year)				
High school or above	766 (30.45%)			
Middle school	981 (38.99%)			
Elementary or below	769 (30.56%)			
Number of siblings (Person)	4.09	1.63	0	15
Women’s income (10,000 CNY/Year)	1.71	1.12	0.00	8.50
Direct cost of child (10,000 CNY/Year)	1.84	1.91	0.33	14.25
Social security (CNY/Month)	122.92	31.23	80.79	177.91
Traditional culture				
Weakly influenced	353 (14.03%)			
Moderately influenced	1,653 (65.70%)			
Deeply influenced	510 (20.27%)			

### Regression results

The regression results of both univariate analysis and multivariate analysis are displayed in Table 3. One can see that all the independent variables are significantly related to desired fertility at 1% level in the univariate analysis in which only one independent variable was included at a time in the regression model. Nevertheless, some of the control variables lost their statistical significance in the multivariate analysis in which all the independent variables were included at the same time.

**Table 3:** Impact of socioeconomic factors on desired fertility: Generalized poisson regression model analysis (N = 2,516).

Variable	Univariate analysis^a^		Multivariate analysis	
	IRR^b^ (95% CI)	*P*-value	IRR^b^ (95% CI)	*P*-value
Constant			3.7095 (1.8887–5.6260)	<0.0001
Age(Year)	1.0199 (1.0157–1.0241)	<0.0001	1.0001 (0.9921–1.0082)	0.9753
Age at first marriage (Year)	0.9490 (0.9362–0.9620)	<0.0001	0.9861 (0.9705–1.0019)	0.0849
Education				
Elementary or below(Ref.)	1		1	
Middle school	0.7704 (0.7133–0.8321)	<0.0001	1.012 (0.9075–1.1283)	0.8309
High school or above	0.8732 (0.8142–0.9364)	0.0001	1.0331 (0.9389–1.1367)	0.5049
Number of siblings (Person)	1.0689 (1.0501–1.0881)	<0.0001	1.0195 (0.9971–1.0424)	0.0880
Women’s income (10,000 CNY/Year)	0.9233 (0.8983–0.9492)	<0.0001	0.9596 (0.9325–0.9875)	0.0490
Direct cost of child (10,000 CNY/Year)	0.9100 (0.8936–0.9266)	<0.0001	0.9707 (0.9459–0.9961)	0.0243
Social security (CNY/Month)	0.9940 (0.9930–0.9950)	<0.0001	0.9963 (0.9951–0.9975)	<0.0001
Traditional culture				
Weakly influenced(Ref.)	1		1	
Moderately influenced	1.4830 (1.338–1.6438)	0.0013	1.165 (1.0366–1.3092)	0.0104
Deeply influenced	1.6711(1.4905–1.8737)	<0.0001	1.183 (1.0286–1.3608)	0.0185

^a^Univariate analysis: Each variable is included separately in regression model

^b^IRR: Incidence rate ratio

As Table 3 shows, age becomes statistically insignificant in the multivariate analysis. Because the age effect could be confounded by the cohort effect, we have performed a sensitivity analysis of the age effect by including age and cohort separately and simultaneously, and our results indicate that neither age nor cohort has a significant impact on desired fertility. Therefore, the regression results do not support our hypothesis for age. However, women's age at first marriage is still statistically significant at 10% level and negatively related to rural women's desired fertility in the multivariate analysis. This is consistent with our hypothesis and supports the argument that couples desiring small families may marry later [36].

In addition to age, education also becomes statistically insignificant in the multivariate analysis. Thus, one could not conclude any effect of education on rural women’s desired fertility. Nevertheless, we would like to point out that only a few women respondents in this survey have a college education and the result could be different for a survey in urban areas.

In the multivariate analysis, the number of siblings is statistically significant at 10% level and positively related to desired fertility, suggesting that rural women with more siblings are likely to have more children in their lifetime. This is consistent with our hypothesis.

As one of the key variables, women's income is significant at 5% level and negatively associated with their desired fertility in the multivariate analysis (0.9596 [95% CI: 0.9325, 0.9875]): additional increase of 10,000 Yuan in women’s annual income will result in 0.04 decrease in desired fertility.

Another key variable, direct costs of a child is also statistically significant at 5% level and negatively related to women's desired fertility in the multivariate analysis (0.9707 [95% CI: 0.9495, 0.9961]): an increase of 10,000 Yuan in direct costs of a child is associated with 0.03 reduction in desired fertility.

The third key variable that has been argued to have a negative impact on rural women’s desired fertility is social security. Our multivariate analysis shows that the amount of social security payment available to rural elderlies in survey districts is statistically significant at 1% level and negatively related to women's desired fertility(0.9963 [95%CI:0.9951 0.9975]): an100 Yuan increase in social security payment would lead to 0.37 reduction of desired fertility on average.

The results of the multivariate analysis also suggest that the degree of traditional cultural influence on women has a significant impact on desired fertility. The desired fertility of those deeply influenced is 1.183 times that of the weakly influenced ([95%CI: 1.02861.3608], *P*<0.05), and of those moderately influenced is about 1.165 times that of the weakly influenced ([95%CI: 1.03661.3092], *P*<0.05). Although traditional cultural influence is still statistically significant and positively related to women’s desired fertility in the multivariate analysis, we would like to point out that the IRR values for both the deeply influenced and the weakly influenced were greatly reduced when we move from the univariate analysis to the multivariate analysis.

The effects of two variables age and education on women’s desired fertility become insignificant and the impact of traditional culture were greatly reduced when all selected variables in univariate analysis are controlled for each other in the multivariate analysis. To assess the robustness of the results of the three key economic variables (women’s income, direct costs of child, and social security coverage to the elderly) and their impacts on the effects of other three variables (age, education and traditional culture), we estimated three additional models by adding three key economic variables to the multivariate regression model with age, age at first marriage, education, number of siblings, and traditional culture one by one (results not shown). Our results demonstrate that the results of three key variables remain almost unchanged, that the significances of both “age” and “education” were mostly absorbed by these two key variables “social security” and “direct cost of child”, and that the reduction of the influence of traditional culture on desired fertility was due to adding women’s income, direct costs of child and social security coverage for the elderly together.

## Discussion and conclusion

Historically, rural women had a much higher fertility rate in China and rural fertility was the main source for the increase of Chinese population, even in the period of stringent family planning policy to purposely control population growth. Nevertheless, this study has found that on average, rural women's desired fertility in Shaanxi, China in 2015 is only about 1.71, assuming no policy restriction on fertility. Such a number is not only below the replacement fertility level of 2.1, but also below 2.01, the average desired fertility reported by Mo's earlier study based on a nationwide survey of rural women in 2002 in China [74], and 1.8, the average desired fertility disclosed by a report to the Shaanxi Provincial Population and Family Planning Commission in 2013 [75]. Therefore, this study adds further evidence to a decreasing trend and a persistently below replacement level of intended fertility in rural China. In addition, this study has explored major socioeconomic determinants of rural women's desired fertility at the current stage of economic development and the results of our regression analysis indicates that important economic factors such as social security coverage for rural elderlies, direct costs of child and women’s income are major determinants of and all negatively related to rural women’s desired fertility. Our findings suggest the following. Firstly, the availability of social security support for rural elderlies may reduce women's perceived benefits of having children, and thus negatively affect women's desired fertility. Secondly, costs of children are a major concern for rural women’s fertility decision, including the direct costs of having children and women's opportunity cost in terms of income forgone during childbearing and childrearing. In addition to these three economic determinants, the results of our study also demonstrate that two social factors, including the number of siblings and the degree of traditional cultural influence are also determinants of but all positively related to rural women’s desired fertility. The finding of positive association between the number of siblings and rural women’s desired fertility adds more empirical evidence to the theory of the positive relationship between family sizes of successive generations and suggests that having a higher number of siblings may increase rural women's desired fertility. Moreover, the finding of positive relationship between traditional cultural influence and women’s desired fertility suggests that traditional culture towards fertility in the form of social norm is, to a certain extent, still influential to rural women's fertility decision. These findings not only add more empirical evidence to the economic theory of fertility, but also have profound implications to China’s population policy.

In the future, as the Chinese economy further develops, rural women would be more and more present in the labor market and have higher income, more pension benefits and increased costs of having children. That is, these three forces that are negatively related to women’s desired fertility would be on a rise. At the same time, as older generation of women exceeds childbearing age, women in childbearing age would have less siblings and weaker influence of traditional culture towards fertility. Thus, the two forces that are positively related to women’s desired fertility would wither in the future. If such a trend continues, there would be a further decline in fertility in rural Shaanxi. Our empirical evidence along with other national and regional studies (e.g., [14, 21, 74, 75]) suggests that China has probably entered an age of persistently low fertility, and that the possibility of a downward spiral in the number of births, known as the low fertility trap hypothesis [76], can be real. It has been suggested that once fertility has fallen below certain levels and stayed there for a certain time, it might be difficult to reverse such a regime change, resulting in accelerated population aging and shrinking [76, 77]. To stop such a demographic change in very low fertility countries, immediate government actions and some policy measures are needed. With the implementation of the one-child policy for more than 30 years, fertility in urban China remains very low [49]. Along with other studies (e.g., [21, 75], this study suggests that desired fertility in rural China has also fallen further below the replacement level. Hence, we argue that any policy restriction on fertility, such as the current two-child policy, may no longer be necessary. Instead, the government should not only abandon restrictions on fertility, but also start to promote women's fertility in order to avoid the adverse consequences of population aging and shrinking caused by too-low fertility. Given the fact that rural women's fertility decisions are sensitive to major economic factors, including social security coverage for the elderly, the direct costs of having children and income, this study recommends that future government programs should include providing healthcare support for childbearing women and also certain childcare support for families with pre-school age children.

Beside its implications to policy-making, we would like to acknowledge that the findings of this study are based on the survey in Shaanxi province alone and cannot be used to make a broad generalization to the national level. Nonetheless, the results of this study may represent a general trend in desired fertility in rural China since the country as a whole shares a centralized policy-making in economic development and family planning, and has a similar pattern of social economic changes. It is expected that what is happening now in Shaanxi with regarding to rural fertility may have occurred in more developed regions such as coastal provinces and would be happening in less developed areas than Shaanxi. For the purpose of making comparisons between provinces in China, to the best of our knowledge, very few studies have been found on socioeconomic determinants of rural fertility in other provinces or at the national level. It is necessary to conduct new studies to understand more about fertility change in China.
